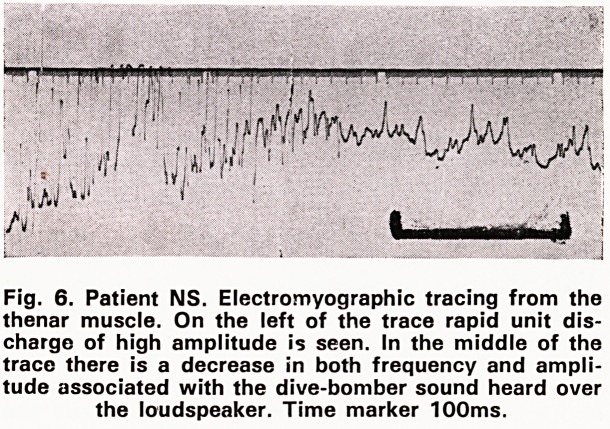# Ocular Myotonia

**Published:** 1975-04

**Authors:** Bryan Ashworth

**Affiliations:** University of Edinburgh, and Neurological Unit, Northern General Hospital, Edinburgh


### Ocular Myotonia

By
Bryan Ashworth, M.D., F.R.C.P., F.R.C.P.E.
University of Edinburgh, and Neurological Unit, Northern General Hospital, Edinburgh

Today, we have heard about several rare diseases and I am about to add another to the pile. This seems to me entirely appropriate to this occasion. Malcolm Campbell, as has been said, was a keen student of natural history. He took a special delight in the recognition of an unusual condition. I should like to add my tribute to his memorial.

Myotonia is a sustained contraction of muscle fibres caused by repetitive depolarisation of their membranes (McComas and Johns, 1969). There is good evidence that it is a disturbance of the muscle itself and it can be demonstrated after the neural connections have been blocked. The three disorders in which myotonia occurs are summarised in Table 1. It has been claimed that they are all essentially the same disease but, in general, each type breeds true in any particular family.

Myotonia can be demonstrated in three ways. As a failure of relaxation after voluntary contraction it is seen best in the grip of the hand, and, as Thomsen pointed out this makes it difficult for the patient to milk a cow. Myotonia can also be shown by percussion of muscle such as the tongue or thenar group. Needle myotonia is a response to insertion of a needle into the muscle and if a concentric needle electrode is used the electrical discharge can be recorded (figure 6).

In dystrophia myotonica there is a combination of myotonia and myopathy. The disability is due mainly to the slow progression of the myopathy. The facial muscles are weak and wasted but not myotonic and clinical tests of eye movement are normal. Ptosis is common and part of the myopathy. The pupil reactions are slow and this has been confirmed by elec-

Table 1.
**Myotonic Syndromes**

| | | | |
|---|---|---|---|
| *Disease* | Dystrophia myotonica | Myotonia congenita | Paramyotonia |
| *Synonym* | Myotonia atrophica | Thomsen's disease | Eulenberg's syndrome |
| *Inheritance* | autosomal dominant | autosomal dominant | autosomal dominant |
| *Clinical features* | Myotonia of tongue, jaw muscles and upper limbs | myotonia generalised | myotonia generalised made worse by cold. |
| | Ptosis, weakness and wasting of trunk, face, and limbs | no weakness hypertrophy of muscles | variable degree of weakness, and wasting |
| *Associated features* | Cataract, frontal baldness, testicular atrophy, low intelligence | — | periodic paralysis a. hypokalaemic b. normokalaemic c. hyperkalaemic |
| *Age of onset* | Infancy, childhood, or early adult | childhood | infancy |
| *Course and prognosis* | Slowly progressive myopathy | symptoms improve with age. Normal life span. | slow progression of limb weakness |

tronic pupillometry (Thompson et al, 1964), but the reason is not clear. Despite the absence of clinical evidence of myotonia of the extraocular muscles the typical electrical abnormality has been reported (Davidson, 1961).

In myotonia congenita (Thomsen, 1876) the main feature is myotonia and may involve any voluntary muscle. Some of the patients report difficulty in moving the eyes and von Graefe's sign was mentioned in several reports (Thomasen, 1948).

Paramyotonia (Eulenberg, 1886) is the rarest of the three conditions and the one usually associated with ocular myotonia. The myotonia is provoked by exposure to cold, and cooling may also precipitate muscle weakness. Muscle wasting and permanent weakness may develop, and there is a link with periodic paralysis. The periodic paralysis may be relieved by giving potassium (hypokalaemic type), or it may be of the type which is provoked by giving potassium (normokalaemic or hyperkalaemic). The ocular features of these syndromes have been reveiwed by Junge (1966).

These relationships are illustrated by two families which we have investigated.

**FAMILY W.**
**Mrs. EW.**

At about the age of 10 she began to have episodes of weakness of the limbs which lasted for a few days. It was difficult to climb stairs and she could not take part in games at school. When she was 17 she had to give up a job in a newsagents shop because of weakness in the legs, but she was able to dance. In her first pregnancy at the age of 39 she became very weak. After a prolonged labour a normal male infant was delivered but the child died some 16 hours later. The muscle weakness improved, but returned during her second pregnancy a year later. There was a miscarriage at 5 months and again the muscle weakness improved. During the third pregnancy at the age of 45 she became very weak and rested in bed most of the time.

At 38 weeks a caesarian operation was performed and the child survived (see BW). Muscle power improved but over the next few years she complained of stiffness and weakness of the muscles. Procaine amide gave considerable relief. After the age of 50 there was a gradual deterioration in muscle power but she was able to get about the house by holding the furniture. She found that in cold weather if she smiled she was unable to relax the muscles of the face. Weakness of the leg progressed and now at the age of 69 she is substantially disabled and uses a wheel chair.

Examination showed good general condition. BP 170/75. Facial myotonia was demonstrated by failure to open the eyes after tight closure (figure 1) and this might take five minutes. The lid-lag sign was present (figure 2). There was a convergent strabismus which appeared to be unrelated to the muscle disorder. The sternomastoid muscles were thin and weak. Muscles of the shoulder girdle and upper limbs appeared normal and the power full. There was wasting of the quadriceps muscles and weakness most marked in the proximal muscles. Tendon jerks were all obtained and plantar responses flexor.
The family history is summarised in figure 3.

Investigation.

The blood count, blood urea, and serum electrolytes were normal. Serum potassium ranged from 3.6 mmol/L to 4.1 blood cholestero! 5.4 mmol/L. Urinary ketogenic steroid excretion normal. Skull radiograph showed evidence of Paget's disease and the serum alkaline phosphatase was 29 King-Armstrong units.

Provocative tests included (1) administration of 150

**Fig. 1. Patient EW. Facial myotonia. Difficulty in opening the eyes after tight closure. This might take as long as five minutes.**

**Fig. 2. Patient EW Myotonic lid-lag. A convergent strabismus is also present.**

**Fig. 3. Pattern of inheritance in family W.**

mmol/L of potassium as potassium chloride. The serum potassium reached 4.3 mmol/L but there was no increase in muscle weakness. (2) administration of 150 grams of glucose and 20 units of insulin. The serum potassium was reduced to 3.1 mmol/L but there was no effect on muscle weakness.

**B.W.** daughter of E.W. was delivered by caesarian section and weighed 7lb. at birth. Very little movement was a "floppy" infant. She walked at 21 months but the gait was never normal. At the age of three she could run but her mother realised at that stage that she had the same condition as herself. She easily became tired and could not join in play with other children. It was difficult for her to walk upstairs and she was often carried into school. At the age of 10 there was an episode of muscle weakness lasting a few days. After this persistent weakness became more marked and slowly increased. At the age of 14 she had frequent falls because of muscle weakness but growth continued normally. At that time her mother first noticed a change in the eyes which seemed to stare.

At the age of 15 severe muscle weakness developed. She could not feed herself or maintain posture in bed. Swallowing was not affected. She remained in this state for about six months and then slowly improved to the point of being able to walk with a frame support.

**Examination** showed facial myotonia and the lid-lag sign. (figure 4). Weakness of facial movements on both sides. Myotonia of the tongue. Neck muscles normal. Weaknesses of the limb muscles, proximal and distal. Tendon jerks all present and plantar responses flexor. Mytonia of the grip of the hand and percussion myotonia of the thenar muscles. No sensory disorder.

**Investigation** showed normal blood count, blood urea, and serum electrolytes; the serum potassium was between 3.3 and 3.8 mmol/L. Creatine phosphokinase 3.5 units (upper normal limit 1.5 units).

Provocative tests included (1) administration of 150 mmol/L of potassium which did not increase the muscle weakness. (2) administration of 150g. of glucose and 20 units of insulin did not affect muscle power.

Serum calcium was 4.5 to 4.7 mmol/L and phosphate 2.2 mmol/L. Calcium gluconate given intraven-

**Fig. 4. Patient BW (daughter of EW) aged 14 years. Myotonic lid-lag.**

ously had no effect on the weakness.

Muscle biopsy taken from the deltoid of EW and quadriceps of BW was reported by Dr. R. M. Norman. In both specimens he found patchy proliferation of muscle nuclei around degenerated muscle fibres, abnormal variation in size of muscle fibres more often due to atrophy than hypertrophy, centrally placed nuclei which were sometimes in chains, and groups of fat cells between fibres. Glycogen stains showed a few large globules in the neighbourhood of fatty spaces but no granules.

Both patients were studied also at Guy's Hospital by Dr. B. McArdle. "investigations were not primarily concerned with potassium metabolism. They had no spontaneous or induced attacks. Muscle potassium levels related to non-collagenous nitrogen were not significantly abnormal."

**FAMILY S.**

The second family has been reported in detail elsewhere (Saunders et al 1968). The father and two sons were affected with myotonia, including myotonia lid-lag and facial myotonia. There was also wasting and weakness of some muscles, particularly the extensors or the forearms. Since the report was published the daughter of one of the affected sons has been seen and found to be a floppy infant.

In one of the subjects paralysis was provoked by

**Fig. 5. Patient NS aged 17 years. (a) forward gaze showing normal relationship of eyelids to eyes and absence of ptosis. (b) downward gaze, following finger, showing myotonic lid-lag. (c) downward gaze; relaxation of myotonia.**

administration of potassium and also by immersion of the legs in cold water. Muscle biopsy showed no fibre necrosis and no vacuolation of the cytoplasm but central nuclei were prominent. The serum creatine phosphokinase was slightly raised. It was noted when a biopsy was taken from the deltoid muscle that the specimen removed showed persistent contraction. The lid-lag sign is illustrated in figure 5.

These two families show the features of Eulenberg's syndrome with myotonia. In the first, there is no definite evidence of abnormality of potassium metabolism but in the second potassium was shown to precipitate weakness.

**Electrical signs of myotonia**

The typical pattern shown on electromyography is seen in figure 6 which is taken from the deltoid muscle of a member of the second family. A rapid discharge of units is seen on the left of the tracing. There is then a diminution in both frequency and amplitude of units and this coincides with the dive-bomber sound heard over the loudspeaker. The essential feature is hyperirritability of the muscle fibre which responds repetitively (Landau, 1952). The rate of discharge of the motor unit may reach 150/second which is more than double the normal rate in response to voluntary contraction.

**Fig. 6. Patient NS. Electromyographic tracing from the thenar muscle. On the left of the trace rapid unit discharge of high amplitude is seen. In the middle of the trace there is a decrease in both frequency and amplitude associated with the dive-bomber sound heard over the loudspeaker. Time marker 100ms.**

**Myotonic features in relation to other signs.**

Mytonia does not occur in response to blinking. When the face is affected it may take several minutes to open the eyes after tight closure. In all these patients this particular symptom was relieved by procaine amide.

The lid-lag sign is useful in ocular myotonia. To demonstrate it the patient is asked to follow the finger upwards and the gaze is held in the extreme upward position for about half a minute. He is then asked to follow the finger downwards and the lid-lag is seen; the appearance is similar to von Graefe's sign and is presumably due to myotonia of the levator palpebrae superioris muscle.

The lid-lag sign has been reported in patients with hyperkalaemic periodic paralysis (van't Hoff, 1962; McArdle, 1962; Gamstorp, 1963) and also with hypokalaemic paralysis (Resnick and Engel, 1967). In these patients the sign is usually present between attacks of paralysis. But the presence of this sign in patients with Thomsen's disease and in patients with Eulenbergs syndrome but no abnormality of potassium suggests that it correlates with myotonia but is unrelated to the state of potassium metabolism.

Facial and ocular myotonia may occur independently. In van't Hoff's family (1962) the lid-lag sign was described but there was no facial myotonia. In the family reported by French and Kilpatrick (1957) there was facial myotonia without the lid-lag sign. In the condition of adynamia episodica hereditaria described by Gamstorp (1956) weakness was provoked by administration of potassium but there was no myotonia and the lid-lag sign was not present.

**SUMMARY**

The myotonic syndromes are reviewed with particular reference to the ocular features. Ocular and facial myotonia are illustrated by reference to two families with Eulenberg's syndrome. In one family there was myotonia but no demonstrable abnormality of potassium metabolism. The other family showed myotonia, muscle wasting, and periodic paralysis of the type provoked by administration of potassium.

The inter-relationships of these features are discussed with reference to cases published previously.

**REFERENCES**

DAVIDSON, S. I. The eye in dystrophia myotonica. Brit. J. Ophthalmology **45**: 183-196 (1961).

EULENBERG, A. Ueber eine familiare durch 6 generationen verfolgbare Form congenitaler Paramyotonie Neurologisches Centralblatt **5**: 265-272 (1886).

FRENCH, E. B. and Kilpatrick, R. A variety of paramyotonia congenita. Journal of Neurology. Neurosurgery, and Psychiatry **20**: 40-46 (1957).

GAMSTORP, I. Adynamia episodica hereditaria. Acta Paediat. Uppsala **45**: supp 108 (1956).

GAMSTORP, I. Adynamia hereditaria and myotonia. Acta Neurologica Scandinavica **39**: 41-58 (1963).

HOFF, W. van't. Familial myotonic periodic paralysis Quarterly Journal of Medicine **31**: 385-402 (1962).

JUNGE, J. Ocular changes in dystrophia myotonica, paramyotonia and myotonia congenita. Documenta ophthalmologica **21**: 1-115 (1966).

LANDAU, W. M. The essential mechanism in myotonia; an electromyographic study. Neurology **2**: 369-388 (1952).

McARDLE, B. Adynamia episodica hereditaria and its treatment. Brain **85**: 121-148 (1962).

McCOMAS, A. and Johns, R. J. Potential changes in the normal and diseased muscle cell. In Walton: *Disorders of Voluntary Muscle* 2nd edition (1969).

McCOMAS, A. J. and Mrozek, K. The electrical properties of muscle fibre membranes in dystrophia myotonica and myotonia congenita. Journal of Neurology, Neurosurgery, and Psychiatry, **31**: 441-447 (1968).

RESNICK, J. S., and Engel, W.K. Myotonic lid-lag in hypokalaemic periodic paralysis. Journal of Neurology, Neurosurgery, and Psychiatry **30**: 47-51 (1967).

SAUNDERS, M., Ashworth, B., Emery, A. E. H., and Benedicz, J. E. G. Familial myotonic periodic paralysis with muscle wasting. Brain **91**: 295-304 (1968).

THOMASEN, E. Thomsen's disease. Universitets forlaget i Arrhus. Denmark (1948).

THOMPSON, H. S., van Allen, M. W., and van Noorden, G. K. The pupil in myotonic dystrophy. Investigative ophthalmology **3**: 325-338 (1964).

THOMSEN, J. Tonsiche Krampfe. Archiv. fur Psychiatrie und Nervenkrankjeiten **6**: 702-718. (1876).

Table 2.

**Myotonic syndromes; ocular and facial features**

| | | | |
|---|---|---|---|
| *Disease* | Dystrophia myotonica | Myotonia congenita | Paramyotonia |
| *Ptosis* | common | absent | absent |
| *Lid-lag sign* | absent | present | present |
| *Myotonia* | tongue, muscles of jaw, but not face or eye muscles | all muscles | all muscles |
| *E.M.G.* | myotonic discharges from ocular muscles (Davidson) | no reports of study of ocular muscles | |
| | myotonic muscles show characteristic pattern | | |
| *Muscle membrane potential* | low* | normal* | low* |

*McComas and Mrozek (1968)

## Figures and Tables

**Fig. 1. f1:**
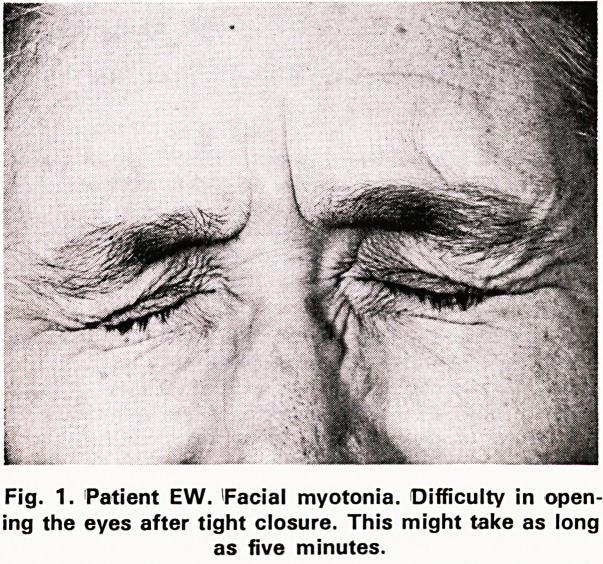


**Fig. 2. f2:**
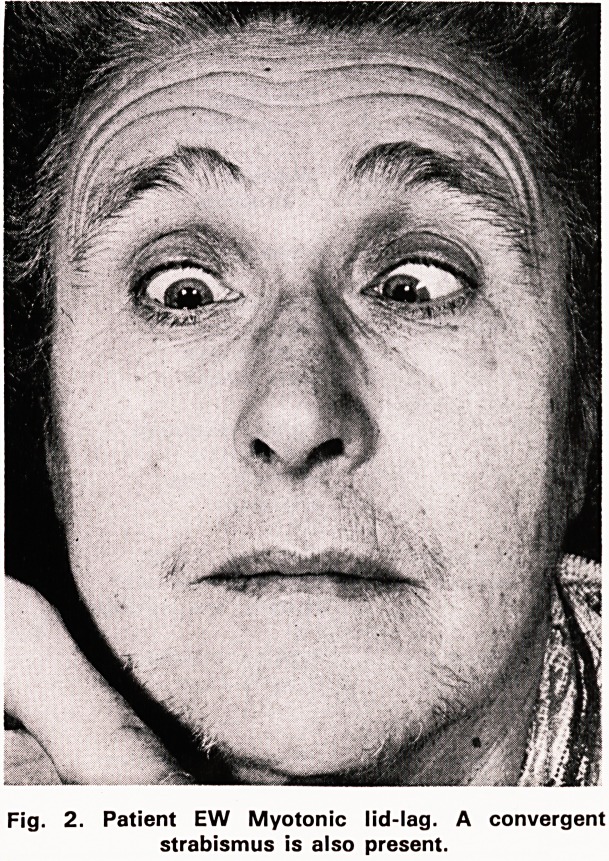


**Fig. 3. f3:**
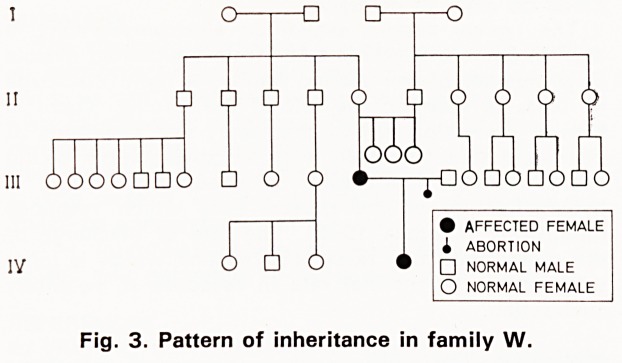


**Fig. 4. f4:**
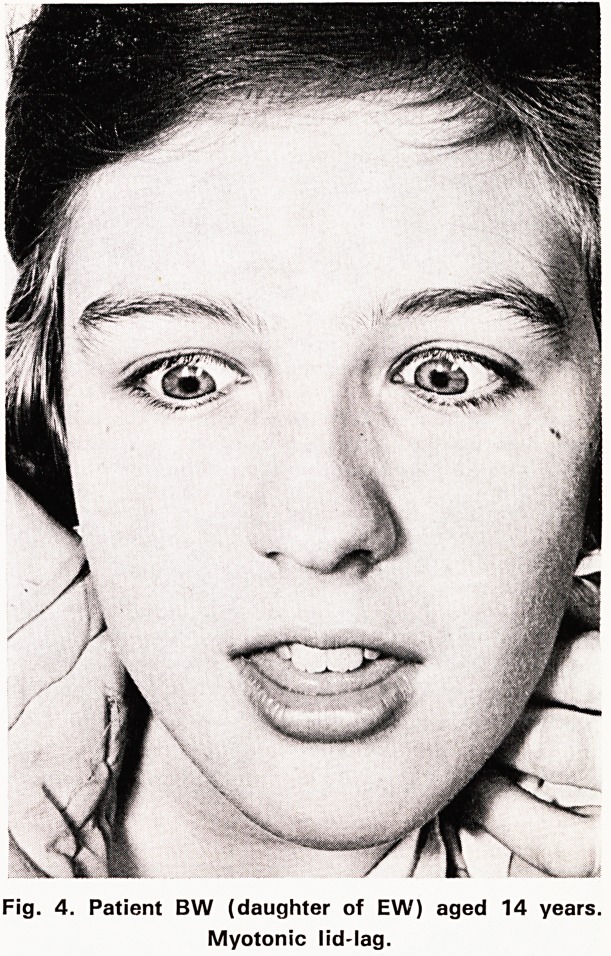


**Fig. 5. f5:**
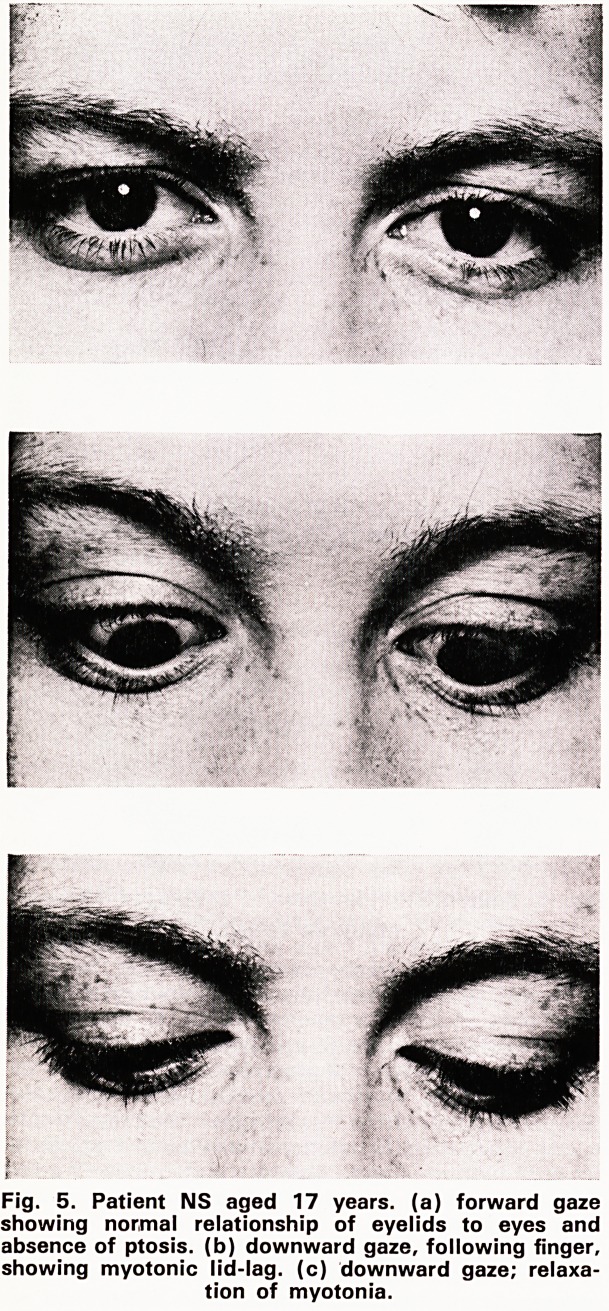


**Fig. 6. f6:**